# Effects of Force Modulation on Large Muscles during Human Cycling

**DOI:** 10.3390/brainsci11111537

**Published:** 2021-11-19

**Authors:** Álvaro Costa-García, Andrés Úbeda, Shingo Shimoda

**Affiliations:** 1 Intelligent Behavior Control Unit, CBS-Toyota Collaboration Center, RIKEN Institute, Nagoya 463-0003, Japan; shingo.shimoda@riken.jp; 2 Human Robotics Group, Physics, Systems Engineering and Theory of Signal Department, University of Alicante, 03690 Alicante, Spain; andres.ubeda@ua.es

**Keywords:** force modulation, neural adaptations, motor control, human cycling

## Abstract

Voluntary force modulation is defined as the ability to tune the application of force during motion. However, the mechanisms behind this modulation are not yet fully understood. In this study, we examine muscle activity under various resistance levels at a fixed cycling speed. The main goal of this research is to identify significant changes in muscle activation related to the real-time tuning of muscle force. This work revealed significant motor adaptations of the main muscles utilized in cycling as well as positive associations between the force level and the temporal and spatial inter-cycle stability in the distribution of sEMG activity. From these results, relevant biomarkers of motor adaptation could be extracted for application in clinical rehabilitation to increase the efficacy of physical therapy.

## 1. Introduction

Voluntary force modulation in humans is the ability to tune the force applied during motion. Given the complexity of the neural system and its non-trivial interactions with the musculoskeletal system, the mechanisms behind voluntary force modulation are still not well understood [1,2,3]. To address these mechanisms, it is necessary to evaluate the factors contributing to muscle force and identify those that enable real-time force modulations. A common opinion within the scientific community is that the origin of muscle force is a result of two main factors: muscle hypertrophy and neural adaptations [1].

Muscle hypertrophy refers to the physical growth of the muscle cells achieved through long-term exercise [4,5], so it cannot be associated with short-term force modulation strategies. Therefore, the origin of real-time force modulation should come from different neural adaptations, which can be roughly summarized into two phenomena: alpha motor neuron firing rate and muscle coordination.

The increase of the alpha motor neuron firing rate is achieved by the enhancement of the neural pathways carrying their activation potentials [6]. For that purpose, the threshold potential of the neurons involved in muscle activation needs to be reduced. Some of the processes that enhance this transmission, like synaptic structural connectivity, require the creation of new neural connections, which cannot be generated or disrupted in real time. However, the threshold potential of a neuron can also be modified in the short-term by activating secondary neural pathways already connected to it. Hereafter, we will use the term “synaptic enhancement” to refer to all those neural processes that facilitate the transmission of potentials through a given neural pathway in real time because they can contribute to real-time force modulation [7,8].

The second neural adaptation associated with the generation of force is muscle coordination. This refers to the recruitment of those neural pathways that coordinate the spatial and temporal activation of a set of muscles generating the most effective force output [9]. Changes in force can also be achieved by efficiently tuning motor control strategies already learned by grown adults. Based on this theoretical background it is reasonable to hypothesize that voluntary force modulation can be achieved by real-time adaptations of muscle coordination and by tuning the synaptic enhancement of the neural pathways involved in muscle fiber activation.

Motor coordination has been studied during human locomotion under different biomechanical constraints [10,11,12,13]. Studies focused on cycling tasks [14,15,16,17] have not found significant changes in motor coordination associated with the modulation of force. A general conclusion is that people use the same modular structure initially learned with small adaptations to different conditions. However, these studies compare the coordinated activation of a large set of muscles, many of them with relatively low force contributions to the movement. Although these small muscles are important for functional motion, their role is related mainly to motor stabilization and postural balance rather than force control [18]. Therefore, the inclusion of these muscles in force-related studies might lead to the disregarding of significant strategy changes produced through larger muscles involved in force modulation. Moreover, a recent study [19] showed that although force modulation and muscle coordination are strongly related in healthy individuals, in stroke patients, there is a clear decoupling between the two parameters. This suggests that other factors contribute to force tuning.

In this work, we study the surface electromyography (sEMG) of the main large muscles responsible for force generation during cycling. Cycling is a well-known bipedal motion [20] that allows for the definition of a controlled experimental environment with reduced motion-induced artifacts, and its force conditions can easily be tuned by modifying the pedaling resistance level. sEMG signals were recorded from the four largest muscles involved in the cycling motion from 10 healthy participants. The contribution of these muscles to each stage of the pedaling cycle were compared across different resistance levels.

The main goal of this work was to study sEMG behavioral changes related to the modulation of force in real time. sEMG signals contain considerable information about the basic neuromuscular processes underlying human motion, and they can be recorded with wearable and increasingly inexpensive devices, which make them an efficient way to study motion. We focus our analysis on two features extracted from the sEMG data: the distribution of sEMG activity among the muscles of interest and inter-cycle variability. In past research, the association of the two parameters with different motor adaptations has been widely examined. For example, Sale et al. [3] extensively reviewed evidence of the neural origins of muscle force, showing that increases in peak force and force rate development are associated with the increased activation of prime mover muscles. In this regard, the study of the distribution of muscle activity across large muscles during different force conditions could elucidate the relationship between their coordination and the modulation of force. The evaluation of sEMG variability has also been used as a tool to infer different neural adaptations [21,22]. Granata et al. found that muscle activity presents twice as much variability among children relative to adult populations, reflecting the increasing stability of trained neural modules [23]. Similarly, Aoyama et al. showed how muscle activity variability decreases for dexterous hand motions [24]. Decreased variability was also recorded for real-time adaptations by Rimini et al. who observed that fast motions present less inter-cycle variability than slow motions [25]. In addition, sEMG stability has been used to measure the loss and recovery of motor coordination after stroke [26]. In light of these previous results, the present study aims to evaluate how this variability is affected by voluntary changes in muscle force.

Finally, the results will be discussed within the exposed theoretical background, exploring their possible connection with the neural adaptations responsible for real-time voluntary force modulation.

## 2. Materials and Methods

### 2.1. Experimental Setup

Ten healthy participants (five women and five men, aged 21.9 ± 0.4 years) participated in the experiments. All participants were right-footed, with no history of motor-related conditions or diseases. All of them were informed about the experimental protocol in advance and signed an informed consent agreement in accordance with the Declaration of Helsinki.

**Figure 1:**
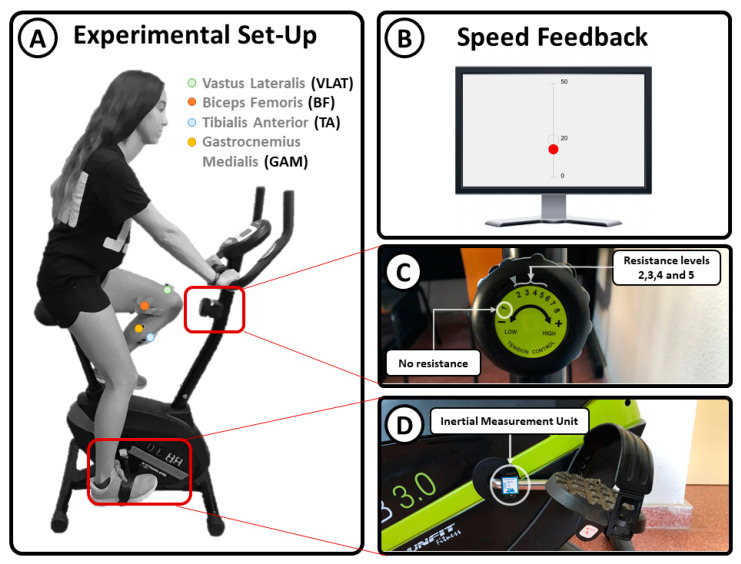
Experimental setup: (**A**) participant position and sEMG electrode location; (**B**) cycling speed feedback; (**C**) resistance level adjustment; (**D**) location of the inertial sensor used to measure cycling speed.

Surface electromyographic (sEMG) data were recorded and digitized at 1500 Hz from the vastus lateralis (VLAT), biceps femoris (BF), tibialis anterior (TA), and gastrocnemius medialis (GAM) using four bipolar electrodes (Noraxon MiniDTS) (Figure 1A) placed on the dominant leg according to the guidelines of the Surface Electromyography for the Non-Invasive Assessment of Muscles project [27]. The experimental environment consisted of a stationary bike (Model RunFit RB 3.0), in which the rotational axis resistance can be switched among different levels: 1, no resistance; 2, very low resistance; 3, low resistance; 4, medium resistance; and 5, high resistance (Figure 1C). The angular velocity was measured with an inertial measurement unit (IMU) placed at the axis of the pedals (Figure 1D). The information recorded from the IMU was used as real-time feedback of the linear speed. This feedback was provided visually to the participants by using a graphical interface showing a red dot moving up and down to represent the speed (Figure 1A).

**Figure 2:**
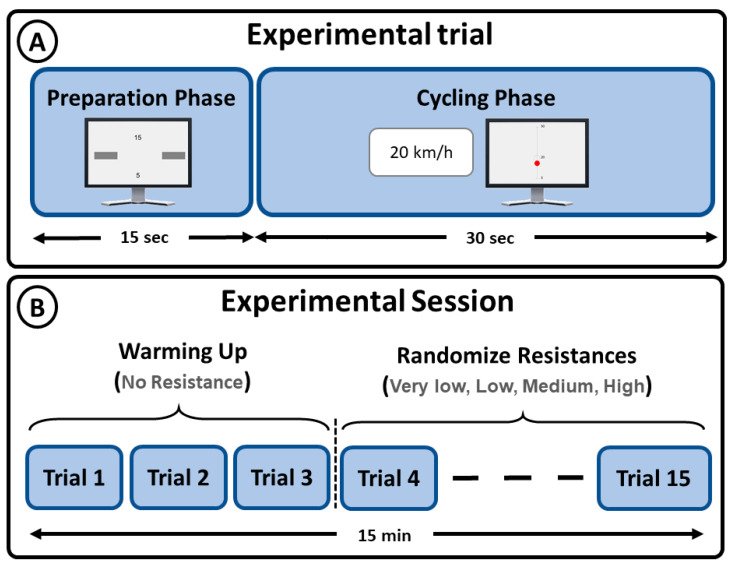
Experimental protocol: (**A**) experimental phases and duration; (**B**) repetitions and total duration of the experiments.

Participants sat on the stationary bike with their knees at a maximum possible extension when the pedal was at its lowest position. They were requested to perform three cycling sessions at a constant speed of 20 km/h without changing their posture. Each session comprised 15 trials of 30 s each (3 trials at each resistance level). Level 1 (no resistance) trials were performed at the beginning of the session as a warmup task. Afterwards, the remaining trials were randomized to avoid temporal bias. Moreover, the randomization was conducted with the constraint that resistance levels 4 and 5, medium and high resistance, respectively, never appeared consecutively to avoid fatigue-related bias. A resting period of 15 s was provided between consecutive trials (Figure 2A). The total time of the experiment was around 15 min per participant (Figure 2B).

### 2.2. Signal Processing and Segmentation

**Figure 3:**
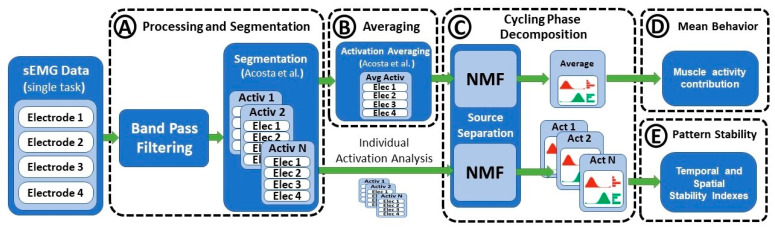
Parameter computation flow: (**A**) filtering processing and segmentation; (**B**) averaging of pedaling cycles; (**C**) extraction of cycling phases; (**D**) estimation of amplitude contribution of each muscle and cycling phase; (**E**) estimation of temporal and stability indexes.

Data normality was assessed by comparing a histogram of the data with a Gaussian normal distribution. Figure 3 shows the general flow of signal segmentation, processing, and parameter estimation. sEMG signals were high-pass filtered at 10 Hz, rectified, and low-pass filtered at 10 Hz prior to the extraction of the muscle amplitude associated with each cycling phase. Moreover, sEMG data were standardized by the median rectified amplitude recorded from the whole set of muscles during each session. This standardization maintained the inter-muscle relative activation ratios and allowed for the comparison of data recorded from different participants. Individual muscle activations were segmented according to the local minima extracted from the envelope of a signal, as described by Costa et al. [28]. This methodology uses the periodical features of the recorded data in order to implement a robust segmentation that maintains the relative spatial and temporal information of the sEMG data without the need for kinematic information (Figure 3A). Such segmentation enables a clearer visualization of the temporal patterns of each motion phase, where ups and downs are directly synchronized with the onset of contributing muscles.

### 2.3. Signal Averaging

Averaging many repetitions of a set of contracting muscles facilitates the extraction of stable amplitude contributions less affected by inter-subject differences. However, even within a single subject, there is significant variation between consecutive motion cycles regarding step length, speed, and inter-muscle temporal and spatial activation timing. Because of these factors, the use of oversimplistic methods for sEMG averaging might result in the loss of relevant motor information. In this work, the activation and deactivation times of each muscle were used as a reference for the definition of an average motion cycle according to the methodology proposed by Costa et al. [28] in which temporal and spatial forms of variation are properly integrated prior to signal averaging (Figure 3B).

### 2.4. Cycling Phase Decomposition

Cycling is divided into two main phases, the power phase (when feet exert force downwards) and the traction phase (when feet move upwards). However, the traction phase is usually strongly supported by the power phase of the opposite leg. For this reason, the pedaling cycle was divided into three phases, the first one to represent the traction phase and the remaining two to represent the power phase.

This phase division was already performed by Hug et al. using the concept of muscle synergies [14]. Muscle synergies are mathematically defined as

(1) M=W×H

(2) $$M\epsilon R^{m\times t};\;W\epsilon R^{m\times n};\;H\epsilon R^{n\times t},$$

where *M* is an *m* × *t* matrix of sEMG data (with *m* being the number of muscles and *t* the number of samples), *W* is an *m* × *n* matrix containing the muscle contributions used to reduce the *m* muscles to an *n*-dimensional space, and *H* is an *n* × *t* matrix containing the *n* phases in which *m* muscles are activated.

Matrices *H* and *W* can be calculated from *M* using a non-negative matrix factorization algorithm [29] by fixing the *n*-dimensionality reduction. In this study, *n = 3* was fixed to decompose the pedaling cycle in three phases (average variance accounted for [VAF] of 96.5%, with a threshold VAF of 95%).

### 2.5. Evaluated Parameters

Four different parameters were evaluated from the decomposition of the pedaling phases. First, the muscle activity associated with each muscle *m* and phase *n* were compared across conditions. The second parameter was the relative contribution of each phase to the total activation period (Figure 3D). The relative contribution can be computed as

(3) $$C_{i}=\frac{\sum_{m=1}^{4}W_{im}\cdot H_{i}}{\sum_{i=1}^{3}\sum_{m=1}^{4}W_{im}\cdot H_{i}}\;,$$

where is *W_im_* is the contribution of muscle *m* during phase *i*, and *H_i_* is the temporal pattern associated with phase *i.*

Finally, the temporal and spatial stability were also computed as the cross-correlation coefficient between the temporal (*H*) and spatial (*W*) patterns extracted from all the motion cycles performed during each resistance level (Figure 3E). This provided two indices ranging from −1 to 1, representing the level of similarity between the set of cycles evaluated within each condition. To study significant differences, paired tests across different resistance levels were computed for each of the parameters (using a Mann–Whitney *U* test).

## 3. Results

**Figure 4:**
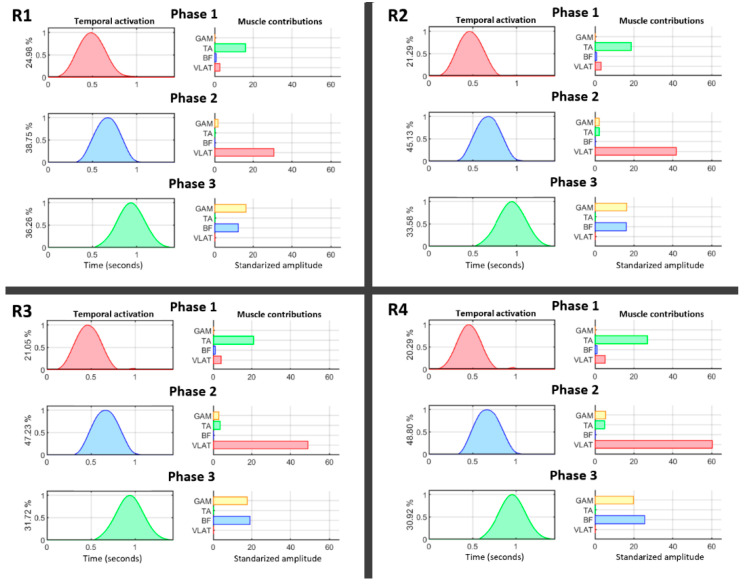
Cycling phase decomposition for all four resistance levels (**R1**–**R4**): phase 1, traction phase; phase 2, initiation of the power phase; phase 3, end of the power phase. The normalized temporal activation (**left**) and the contributions of muscles (**right**) are shown for each phase. The percentages shown along the *y*-axes of the temporal activation graphs represent the relative contribution of each phase to the total motion cycle.

Figure 4 summarizes the behavior of temporal and spatial muscle activations for all four resistance levels. Temporal patterns (right) are represented together with their relative muscle contributions (left). Temporal activations were normalized by their maxima, and this value was transferred to the muscle contributions (right) to maintain the same relative amplitude between them. The motion was divided into three phases (VAF > 95%) showing the activation of TA, followed by VLAT, and, finally, a coactivation of GAM and BF during the end of the pedaling cycle. A deeper analysis of this behavior showed that there was a decrease in TA activity only under the highest resistance level. However, VLAT showed an initial increase from the beginning, and it was the most active muscle when the resistance level was increased (*p* < 0.05, Mann–Whitney *U* test). Another interesting finding is the shift between BF and GAM contributions. BF was less active at lower resistance levels but gradually dominated when dealing with higher force efforts (*p* < 0.05, Mann–Whitney *U* test).

**Figure 5:**
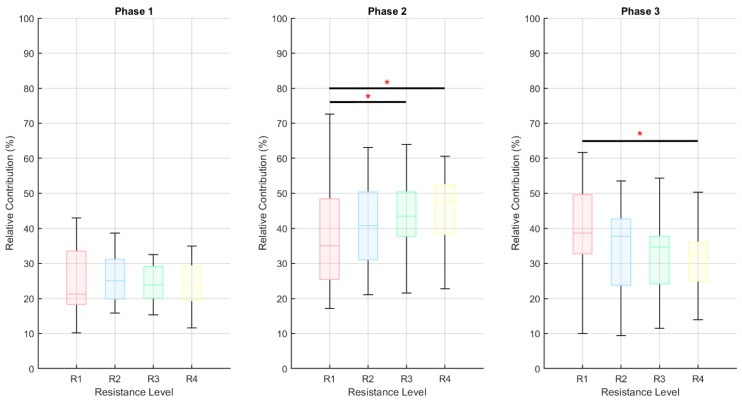
Comparison of the relative contribution of each phase depending on resistance level (*, *p* < 0.05). The relative contributions were computed based on Equation (3), and they represent the percentage of the total signal amplitude generated by each motion phase.

Figure 5 shows the phase-relative contribution changes across conditions. A significant decrease has been found in phase 2, when VLAT was more active, while phase 3 decreased in proportion, showing a smaller influence of GAM and BF when the resistance level was increased (*p* < 0.05, Mann–Whitney *U* test). Phase 1, which is mostly related to TA activity, was relatively stable across conditions.

**Figure 6:**
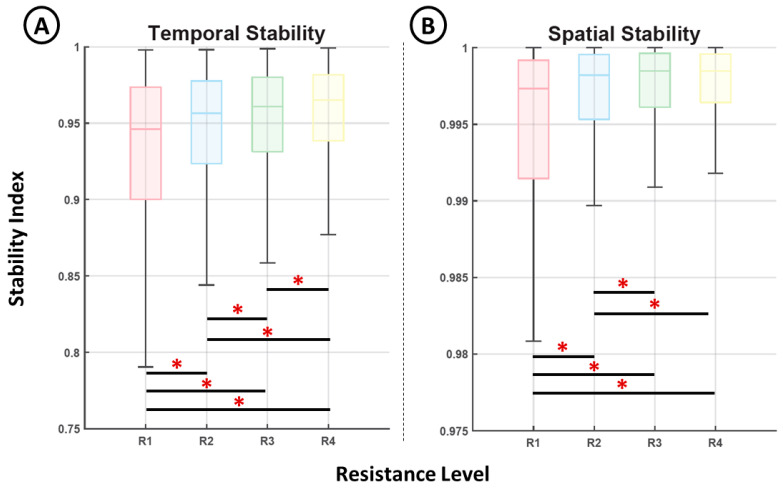
Comparison of the temporal stability index (**A**) and the spatial stability index (**B**) for each resistance level (*, *p* < 0.05).

Figure 6 shows the representation of the stability indices computed from the temporal (Figure 6A) activation data and the associated muscle contributions (Figure 6B) for each motion phase and resistance level. Both indices show a clear increase in their median values for higher resistance levels. This increase, although constant, was limited to a small range (from 0.78 to 0.99 in the case of the temporal stability and 0.9981% to 0.9999% in the case of spatial stability). In addition, all distributions exhibited significant differences (*p* < 0.05, Mann–Whitney *U* test) except for the two spatial stability indices computed for resistance levels 3 and 4.

## 4. Discussion

Although previous studies did not find significant differences in muscle coordination across a range of pedaling constraints [14,15,16,17], our results showed that real-time force modulation is significantly associated with changes in the sEMG distribution of the main muscles involved in force generation.

This is clearly shown in the functional shift reported between the GAM and BF (Figure 4) under increasing levels of resistance. Although both muscles play an active role during the last stage of the power phase of pedaling, under increasing force demands, the activation of BF is dominant. Moreover, during the power phase, the VLAT, which is directly connected to this phase, increases its contribution under high resistance conditions (Figure 4); this is the muscle initiating the power phase (phase 2) and its inertial motion supports the end of the phase (phase 3). These results suggest that a quick achievement of a critical amount of force at the beginning of the power phase becomes more relevant to maintaining a constant pedaling speed under higher resistance conditions. The motor adaptations indicated by these results demonstrate how changes in the coordination of large muscles represent a significant factor in the real-time modulation of force.

In addition, our results reveal positive associations between the force level and the temporal and spatial inter-cycle stability in the distribution of sEMG activity across the evaluated muscles. This outcome could be a result of the more stable recruitment of the neural modules associated with higher force motions, which aligns with previous research showing increased muscle stability during dexterous [24] and fast [25] motions. Although this interpretation is less certain because of the lack of direct recordings from the neural pathways, the potentiation of the neural pathways should at least be considered as a possible mechanism that explains this result. From the known neural processes that can be tuned in real time, synaptic enhancement could explain the relative increase in the dominance of the recruited neural modules.

Although the outcome of the present research has been discussed from a neurophysiological perspective, the metrics evaluated are based on observable changes in muscle activity, and no data were recorded directly from the brain or the spinal cord. Additional neurophysiological data should be used in future research to establish a stronger connection between results like those presented herein and the neural adaptations responsible for real-time force modulation. The inclusion of musculoskeletal modeling should also be considered in order to evaluate possible biases related to biomechanical constraints. Moreover, the results presented in this paper should be further validated across a variety of tasks and with a larger set of muscles.

One relevant application of the assessment of motor adaptations by means of the electrophysiological analysis is the establishment of meaningful biomarkers of motor recovery. To date, many studies have investigated the use of other parameters such as kinematic or dynamic information in motor rehabilitation tasks as a means of monitoring therapeutic outcomes [30]. The use of surface electromyography has great clinical potential in the monitoring of neuromuscular pathologies and the evaluation of treatments [19,31,32,33,34,35]. The process behind the decoupling between force modulation and motor coordination reported for patients suffering from motor diseases is still unclear [19]. However, it is known that, after stroke, there is a short time window in which motor recovery skills are enhanced. Therefore, early malfunction detection followed by the selection of an appropriate rehabilitation strategy is a pressing requirement for efficient recovery. There are plans to expand the protocol and methodology introduced in this work to clinical rehabilitation by recording the sEMG data from patients with motor diseases such as stroke and spinal cord injury. The goal of this future research will be to evaluate deviations in real-time force modulation mechanisms using the data recorded from healthy individuals as ground truth. This comparison will be used to determine which biomarkers are the most relevant in order to detect malfunctions in this mechanism and how they can be used for the selection of appropriate rehabilitation strategies.

## 5. Conclusions

This work examined the motor adaptation of the large muscles responsible for force generation during a cycling task across four different resistance conditions. We found significant differences in the sEMG signal spatial distribution as a response to changes in force demands affecting mainly the power phase of the cycling motion. Moreover, the inter-cycle temporal and spatial sEMG distributions show increasing stability under higher force constraints. These results were discussed from a neurophysiological view, establishing a possible connection between them and the neural adaptations responsible for real-time muscle force modulation. Through this work, we aim to develop a set of biomarkers that will allow for the quantification of real-time force modulation skills, with the future goal of applying them in clinical rehabilitation.

## Data Availability

The data presented in this study are available on request from the corresponding author. The data are not publicly available due to the private policy of the ethics committee under which experiments were conducted.
